# Mapping Neuroanatomical Heterogeneity of Brain Aging Within a Clinically Defined Aging Reference Cohort

**DOI:** 10.3390/bioengineering13070844

**Published:** 2026-07-22

**Authors:** Yanxue Li, Hongjian Gao, Lan Lin, Min Xiong

**Affiliations:** 1 Department of Biomedical Engineering, College of Chemistry and Life Science, Beijing University of Technology, Beijing 100124, China; liyanxue@emails.bjut.edu.cn (Y.L.); gaohongjian@bjut.edu.cn (H.G.); 2 School of Science, North China University of Technology, Beijing 100144, China

**Keywords:** brain aging, deep learning, structural MRI, heterogeneity, influencing factors, UK Biobank

## Abstract

Brain aging exhibits substantial interindividual heterogeneity, yet separating aging-related neuroanatomical variation from pathological influences remains methodologically challenging. To address this issue, we constructed a Clinically Defined Aging Reference (CDAR) cohort from the UK Biobank by excluding individuals with overt clinical pathology and applied the Surreal-GAN framework to characterize latent patterns of age-associated structural variations. A total of 26,251 participants were included. The model identified two co-occurring dimensions of brain aging, referred to as R_1_ and R_2_, that were stable across subsamples (R_1_: r = 0.873, R_2_: r = 0.953) and remained consistent when refitted separately in males and females (female: r = 0.792, male: r = 0.818). R_1_ was characterized by widespread gray matter reduction involving cortical, subcortical, and cerebellar regions and was associated with broadly poorer cognitive performance, adverse lifestyle profiles, metabolic and inflammatory alterations, and age-related diseases. R_2_ exhibited relative preservation of subcortical structures together with widespread preservation of cortical surface area and more selective differences in cortical thickness. Compared with R_1_, R_2_ showed weaker associations with cognition and peripheral physiological measures but retained associations with cardiovascular-related outcomes. These findings suggest that brain aging within a clinically defined aging reference cohort may involve multiple partially dissociable neuroanatomical dimensions rather than a single pattern, providing an operational reference for studying aging-related structural heterogeneity under reduced clinical confounding.

## 1. Introduction

Aging is a complex biological process characterized by the progressive accumulation of molecular and cellular damage throughout the lifespan, which the organism is unable to adequately repair, ultimately leading to the loss of physiological function [1,2]. The brain is particularly sensitive to the effects of aging, manifesting in both structural and functional alterations with advancing age. Common changes include brain atrophy, reflected in reductions in gray matter (GM) volume and cortical thinning [3], decreased white matter (WM) integrity and volume [4], and aberrant functional connectivity [5]. These changes render the brain increasingly vulnerable to a spectrum of age-related neurological disorders [6,7]. While research often focuses on these pathological trajectories to identify early markers for neurodegenerative diseases, understanding the fundamental heterogeneity of the normal aging process itself remains a prerequisite for accurately distinguishing between healthy senescence and preclinical pathology [8]. This distinction is critical because brain aging cannot be adequately described as a uniform or single dimension. Instead, it exhibits substantial heterogeneity across individuals, shaped by genetic predisposition, environmental exposures, and lifestyle factors [9]. This heterogeneity manifests not only in the rate of global atrophy but also in the spatial distribution of GM changes, with region-specific and nonlinear patterns observed across the lifespan. Emerging evidence further suggests the coexistence of distinct aging phenotypes, such as accelerated versus resilient aging [10], even among ostensibly healthy individuals. This raises a critical challenge: while the biological diversity of brain aging is increasingly recognized, it remains insufficiently modeled and poorly captured by existing analytical frameworks.

In recent years, many studies have leveraged neuroimaging data to quantify brain heterogeneity. The emergence of artificial intelligence has introduced novel perspectives by integrating AI methodologies with neuroimaging data [11,12]; nevertheless, fundamental advances in understanding normative brain aging remain elusive. A substantial body of prior work has focused primarily on disease-related brain heterogeneity, revealing diverse atrophy patterns and heterogeneous trajectories within patient populations [13,14]. In contrast, the characterization of heterogeneity in normative brain aging has remained in its early stages. Converging evidence indicates that even among healthy individuals, the process of cerebral aging is modulated by genetic architecture, environmental exposures, and lifestyle variables [15], resulting in brain states that appear either older or younger than chronological age. A subset of studies has approached heterogeneity in brain aging through the lens of accelerated aging. For example, Eavani et al. [16] employed the brain age gap (BAG) metric to stratify participants into normal and accelerated aging groups, within which a semi-supervised Mixture of Experts (MOE) algorithm further delineated five distinct accelerated aging patterns. Similarly, Liu et al. [17] utilized the BAG metric in the considerably larger UK Biobank (UKB) dataset and applied the HYDRA framework to dissect heterogeneity within the accelerated aging cohort, revealing three distinct subtypes of brain aging. However, these methods typically rely on “hard-clustering” paradigms, which fail to capture the biological reality that multiple aging-related patterns often coexist and evolve simultaneously within the same individual.

Against this backdrop, generative adversarial networks (GANs) [18], as an exploratory analytical framework, have shown unique promise. By leveraging their powerful capacity for non-linear feature learning and latent space disentanglement, GANs can effectively separate inherent structural heterogeneity from confounded imaging data. Preliminary efforts, such as Smile-GAN and Surreal-GAN [19,20], have applied these generative frameworks to resolve brain heterogeneity. Specifically, Smile-GAN learned a mapping from the control group to the patient group, disentangling confounding factors unrelated to the disease and thereby revealing the expression probabilities of various brain atrophy patterns [21]. Building on this, Surreal-GAN [20] further introduced continuous latent variables to characterize the manifestation of coexisting atrophy patterns, effectively capturing the gradual progression from mild to severe pathological states. By incorporating a Gaussian copula to model interdimensional correlations, it further refined the representation of intricate comorbidity patterns within diverse cohorts [22]. By explicitly separating normal aging trajectories from disease-specific topographic variations, such disentanglement models effectively conceptualize brain alterations as distinct, parallel processes. However, these methodological advances inevitably push a broader question to the forefront of brain-aging research: how should latent neuroanatomical variation be conceptualized when aging-related and disease-related processes coexist within the same population? Addressing this requires reconciling fundamentally different theoretical frameworks regarding the relationship between physiological senescence and neuropathological progression.

In neuroimaging research, representing the latent structure of brain aging involves distinct conceptual frameworks. One representative perspective conceptualizes normal aging and pathological progression as a continuous, overlapping spectrum sharing common biological substrates [23], referred to as the Health-to-Disease Continuum (HDC) framework. However, in standard heterogeneous cohorts, statistical variance driven by subclinical pathological processes often overlaps with typical senescence signals, making the empirical boundary between healthy aging and early pathology difficult to delineate [24]. Variance structures extracted from mixed populations tend to reflect a blended representation of chronological aging and unrecognized clinical processes, which limits the interpretive clarity of derived associations [25]. An alternative position holds that, although aging and pathology are not biologically independent, their contributions to neuroanatomical variance can be methodologically dissociated under appropriately controlled study designs [26,27,28,29]—we term this the Dissociable Aging-Pathology Hypothesis (DAPH).

Although these two hypotheses provide theoretical frameworks for understanding heterogeneity in brain aging, two critical gaps remain in current research. First, most generative modeling studies have treated healthy control groups as relatively homogeneous reference baselines, without explicitly examining whether structured heterogeneity exists within a clinically screened reference cohort itself. Second, it remains unclear whether, after reducing the confounding influence of overt disease, brain aging manifests as multiple dissociable dimensions reflecting different biological processes, rather than a single global spectrum of morphological variation. To address these gaps, we constructed a Clinically Defined Aging Reference (CDAR) cohort by systematically excluding overt clinical pathology and applied the validated Surreal-GAN framework to characterize latent patterns of brain aging within this low-confounding reference space. Rather than developing a new representation-learning framework, our objective was to determine whether constraining disease-related variance changes the latent organization of brain aging and, if so, whether the recovered latent dimensions exhibit dissociable neuroanatomical, cognitive, and systemic phenotypic profiles. Specifically, we asked whether brain aging within the CDAR cohort remains multidimensional after overt disease-related variance is constrained, and whether the resulting latent dimensions display distinct anatomical, cognitive, and peripheral phenotypic characteristics.

The remainder of this paper is organized as follows. Section 2 delineates the methodological framework, including imaging data preprocessing, the core experimental design, and hyperparameter optimization for the model. Section 3 provides a comprehensive presentation of the empirical results, encompassing the determination of optimal model parameters, the characterization of the two distinct brain aging patterns, and an in-depth analysis of their correlations with multidimensional phenotypic measures and disease risk. Section 4 discusses the findings in the broader context of neuroimaging research on brain aging, while also addressing the clinical implications, strengths, future research directions, and inherent limitations of this study. Section 5 concludes the paper by synthesizing the key contributions.

## 2. Materials and Methods

### 2.1. Data and Preprocessing

#### 2.1.1. Study Population

Data for this study were sourced from the UKB cohort, which comprises over 500,000 volunteers from across the United Kingdom aged 40 to 69 years [30,31]. The UKB imaging extension [32], initiated in 2014, represents one of the largest and most comprehensive neuroimaging datasets to date. It incorporates six imaging modalities, facilitating in-depth characterization of brain structure, microstructure, and functional activity. The UKB study was conducted under ethical approval from the North West Multi-Centre Research Ethics Committee (REC reference 11/NW/0382). This specific research was performed under an approved UKB application (68382).

#### 2.1.2. Study MRI and Imaging-Derived Phenotypes (IDPs)

Neuroimaging data were collected using a uniform 3.0 Tesla Siemens Skyra platform (Siemens Healthineers, Erlangen, Germany). This multi-modal protocol utilized T1-weighted sequences (TR = 2000 ms, TE = 2.01 ms, TI = 880 ms, flip angle = 8°) for high-resolution anatomical mapping. To ensure data consistency, the UKB team implemented a centralized processing pipeline that converts raw signals into validated IDPs. These IDPs quantify multifaceted dimensions of cerebral morphology. As of late 2022, the repository released processed outputs for approximately 42,000 participants. Within this framework, the T1-weighted modality contributes multiple types of IDPs, spanning regional volumes, cortical thickness, and cortical surface metrics. Technical specifications regarding acquisition parameters and post-processing architectures are documented in the official UKB manuals (https://biobank.ctsu.ox.ac.uk/crystal/crystal/docs/brain_mri.pdf, accessed on 20 September 2025).

To ensure structural integrity and methodological rigor, T1-weighted structural MRI data were processed following the standard UKB imaging pipeline using the FMRIB Software Library (FSL, version 5.0.10). Initial preprocessing involved reorientation to the MNI standard, robust FOV reduction to remove neck tissue, and bias-field correction to mitigate B1 inhomogeneity. For regional brain quantification, 139 imaging-derived phenotypes (IDPs) representing GM volumes were extracted, providing comprehensive coverage of cortical GM (96 regions), subcortical structures (15 regions), and the cerebellum (28 regions), based on a multi-atlas framework integrating the Harvard–Oxford cortical and subcortical atlases with the Diedrichsen cerebellar atlas. For each participant, T1-weighted images were linearly registered using FLIRT and subsequently non-linearly aligned to the MNI152 1 mm template using FNIRT to estimate forward and inverse deformation fields. In parallel, tissue-type segmentation was performed using FSL-FAST to generate voxel-wise Partial Volume Estimation (PVE) maps for GM, WM, and cerebrospinal fluid (CSF). To preserve subject-specific anatomical precision, atlas-defined ROIs were transformed from MNI space back into each participant’s native space using the inverted warp fields. Regional GM IDPs were then quantified by summing GM PVE values within each native-space ROI, yielding a standardized and anatomically individualized representation of whole-brain morphology for subsequent model training.

Cortical thickness and surface area measures were obtained from the UKB IDPs, which were generated using the standardized FreeSurfer [33] processing pipeline. Briefly, T1-weighted images underwent automated preprocessing including intensity normalization, skull stripping, white and pial surface reconstruction, topology correction, and tissue segmentation. Cortical morphometric measures were subsequently computed on the reconstructed cortical surfaces. Regional anatomical parcellation was performed according to the Desikan–Killiany–Tourville (DKT) atlas, which subdivides the cerebral cortex of both hemispheres into 62 anatomically defined regions. For each parcel, FreeSurfer estimated mean cortical thickness as the average distance between the white matter and pial surfaces, whereas cortical surface area was calculated from the tessellated cortical mesh and aggregated within each region. Regional mean cortical thickness and surface area were then extracted for each DKT parcel. To enable a cross-pipeline consistency check, the DKT-derived measures were excluded from model training. Specifically, the Surreal-GAN model was trained exclusively on 139 whole-brain GM volume IDPs derived from the FSL-FAST pipeline, whereas the DKT-based cortical thickness and surface area measures were reserved for downstream characterization of the identified aging patterns. This cross-pipeline design enabled evaluation of aging-related morphological signatures using a complementary surface-based morphometric representation. In total, this yielded 139 volume-based IDPs from FSL for model training, and 124 DKT-based IDPs (62 thickness + 62 surface area) from FreeSurfer for downstream characterization.

#### 2.1.3. Non-Imaging-Derived Phenotypes (Non-IDPs)

To complement the neuroimaging data, a comprehensive set of non-IDPs was derived from UKB assessment records, spanning sociodemographic factors, lifestyle behaviors, and physical health status, and biological samples collected from the baseline recruitment through the imaging follow-up. To investigate potential factors influencing brain aging, we performed a systematic curation of variables identified in existing gerontological literature as relevant to the aging process, resulting in a finalized set of 262 variables. These variables were harmonized and organized into seven functional domains: cognitive function (26 variables); blood assays (61 variables); physical measures (29 variables); lifestyle and Environment (86 variables); psychosocial factors (16 variables); sociodemographic characteristics (34 variables); and early-life factors (10 variables).

All selected variables were rigorously classified into four distinct data types for statistical modeling based on their original UKB encoding schemas and empirical distributional: continuous, count, binary, and ordinal categorical. Special numerical codes representing non-responses (e.g., “Prefer not to answer” or “Do not know”) were recoded as missing values or realigned to ensure that all quantitative scales possessed consistent directionality and biological interpretability. To maximize the utilization of the valuable neuroimaging dataset and maintain optimal statistical power, a pairwise available-case approach was adopted for the downstream associations, rather than restricting the analysis to an overly conservative intersected complete-case subset. Specifically, missing values were handled using a complete-case approach at the level of each analysis, such that each model was fitted using the subset of participants with no missing data on the variables required for that particular model. This strategy is widely standard in large-cohort epidemiological studies (such as the UKB) where missingness is heavily variable across distinct sub-questionnaires and biomarkers. To ensure transparency, the specific effective sample size (N) for each regression model is explicitly reported alongside its corresponding effect size.

### 2.2. Neuroanatomical Pattern Discovery via Surreal-GAN

#### 2.2.1. Model

We employed Surreal-GAN [22], a weakly supervised generative adversarial framework, to characterize the spatially distributed and temporally progressive patterns of brain aging. Unlike traditional regression models, Surreal-GAN effectively decomposes the inherent neuroanatomical heterogeneity of the aging brain into multiple latent dimensions (R-indices), quantifying the expression level of distinct structural transformation patterns. The architecture comprises three integrated components: a Generator G, a Discriminator D, and an Inverse Mapping Network I. Specifically, G utilizes a non-linear additive structure to transform reference (REF) inputs into synthetic target-like samples by applying diverse morphological transformation patterns conditioned on latent variables. D is trained to differentiate between TAR samples and G-generated synthetic data. Crucially, the Inverse Mapping Network—consisting of a decomposer and a reconstructor—infers the latent variables by decoupling complex structural variations back into independent aging dimensions. This ensures that each identified pattern is both identifiable, ultimately providing a continuous, low-dimensional representation of brain aging.

#### 2.2.2. Study Design

To meet the intrinsic requirements of the Surreal-GAN architecture—specifically, the need for distinct distributional divergence between the reference (REF) and TAR groups to capture non-linear structural transformations—we implemented a discrete age grouping strategy. The REF group was defined as participants aged ≤55 years, representing a younger reference distribution, while the TAR group comprised those aged ≥65 years, representing a later-life distribution. These thresholds were selected based on three considerations: (i) the literature indicates that the transition from midlife to late life (approximately 55–65 years) is characterized by accelerated structural brain changes, supporting the use of ≤55 as a pre-aging baseline and ≥65 as a later-life comparison group [34]; (ii) the Surreal-GAN framework requires sufficient distributional divergence between the REF and TAR groups to learn structural change patterns, and the 10-year age gap between these thresholds provides this level of contrast; and (iii) the UKB sample is predominantly middle-aged—adopting more extreme cutoffs (e.g., ≤50 vs. ≥70) would substantially reduce sample size and compromise model training stability. This choice therefore reflects a pragmatic trade-off among distributional contrast, sample adequacy, and biological plausibility, rather than an attempt to identify a universally optimal age cutoff.

Based on the DAPH, this study constructed a CDAR cohort by rigorously excluding overt clinical pathology, thereby providing a data foundation for the model to learn neuroanatomical patterns from a clinically defined operational reference cohort. Exclusion codes for the CDAR cohort were selected based on four principles: (1) conditions that directly affect brain structure; (2) diseases with established cerebrovascular consequences; (3) chronic systemic diseases known to involve sustained neuroinflammation; and (4) major central nervous system insults. Conditions meeting any of these four criteria were excluded based on ICD-10 coding records at any time point up to and including the imaging visit (see Supplementary Material, Table S1 for exclusion codes). It should be noted that CDAR, as a clinically defined operational reference, cannot fully exclude subclinical pathological burden. Therefore, it represents an operationally defined reference cohort that minimizes variance arising from manifest clinical conditions. All other participants were classified as non-CDAR individuals (the general unscreened population). The non-CDAR REF group was excluded as it did not align with aging patterns in a clinically screened reference cohort. Following these criteria, 26,251 participants were included. Accordingly, the CDAR cohort should be interpreted as a pragmatic operational reference defined by these exclusion principles, rather than as a universally valid “healthy aging” baseline. Although this design minimizes variance attributable to manifest clinical conditions, residual latent pathology may still be present.

**Figure 1:**
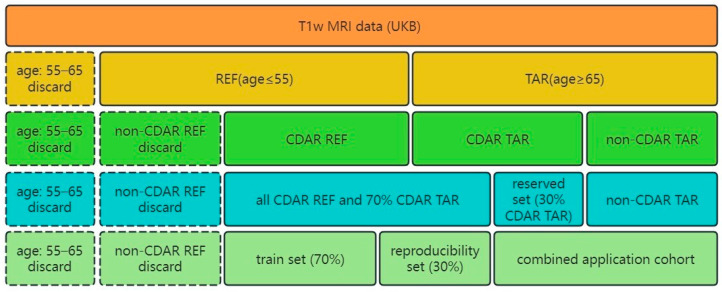
Two-stage stratification and data partitioning strategy.

Individuals aged 55–65 years were excluded from both the REF and TAR groups during model training. The model was trained on two age-stratified anchor distributions representing middle age and older age strata, rather than on a continuous age spectrum. The R-index was defined as the projection of individual brain morphology onto a latent axis learned from contrastive modeling between REF and TAR groups. It reflects the relative expression of age-associated morphological variation along this axis. This latent axis represents a data-driven discriminative dimension separating the two anchor age distributions. As illustrated in Figure 1, we employed a stratified random sampling approach to partition the study population. To ensure unbiased evaluation, 30% of the CDAR-TAR group (n = 3398) was set aside as the reserved set, which was excluded from the initial model development phase. The remaining CDAR cohort—comprising the entire CDAR-REF group and the remaining 70% of CDAR-TAR group—was further partitioned, with 70% allocated to the training set (n = 9194), and utilized to train the model for capturing brain aging patterns, while the remaining 30% (n = 3940) was designated as the reproducibility set and used to independently retrain a new Surreal-GAN model to evaluate the cross-sample reproducibility of the identified aging patterns. Due to the weakly supervised nature of Surreal-GAN, no independent validation set was established. Instead, hyperparameter selection and optimal model determination were achieved by repeatedly training the model solely within the training framework and maximizing between-model consistency (*R*_indices_corr_). Throughout this process, model reproducibility served as the proxy metric for conventional generalization error. Following model development, the optimal model was applied to the combined application cohort (n = 13,117), formed by merging the reserved set (n = 3398) with the non-CDAR TAR participants (n = 9719), to characterize the neuroanatomical features of the aging patterns and to perform downstream association analyses with various phenotypic variables and disease risks. It should be noted that, as a clinically defined reference, the CDAR cohort cannot fully rule out undetected preclinical pathology (e.g., silent amyloid or tau deposition), a limitation acknowledged in the Introduction and inherent to this design.

#### 2.2.3. Model Training and Hyperparameter Optimization

To minimize the influence of confounding variables, imaging features were first adjusted for sex and intracranial volume (ICV) using linear regression parameters derived exclusively against the REF group. These features were subsequently residualized and standardized relative to the REF baseline to achieve a mean of 1 and a standard deviation of 0.1 (i.e., variance = 0.01), resulting in a 139-dimensional feature vector per participant that serves as the input to Surreal-GAN. The model was trained on the curated cohort of clinically defined individuals, with the REF group and TAR group defining the baseline and target distributions, respectively. We performed a comprehensive grid search to identify the optimal configuration for three critical hyperparameters: the number of latent patterns (*C* in [2, 5]), the orthogonality constraint (*λ* in [0.05, 1.6]), and the change magnitude regularization (*γ* in [0.1, 8]). Other secondary hyperparameters were fixed at their default settings (*α* = 0.02, *κ* = 80, *ζ* = 80, *μ* = 500, *η* = 6). For each hyperparameter combination, the model was trained for 30,000 epochs with a batch size of 300. At intervals of 1500 epochs, a checkpoint was saved if both the monotonicity loss and the reconstruction loss were below 0.003. Each hyperparameter combination was repeated 20 times, and the combination yielding the highest *R*_indices_corr_ was selected as the optimal configuration. Subsequently, using this selected configuration, we conducted 40 independent training runs to assess training stability, and the final model was chosen as the run with the highest *R*_indices_corr_. Because *R*_indices_corr_ inherently favors lower-dimensional solutions, model selection was further complemented by an independent nested model comparison (Section 2.3.1). This additional analysis was designed to determine whether the selected solution captured meaningful neuroanatomical variation beyond chronological age, rather than merely reflecting the metric’s intrinsic preference for lower-dimensional representations.

Furthermore, to rule out potential underestimation of dimensionality, we trained higher-dimensional models using the same optimal hyperparameter configuration. The resulting dimensions were compared with the chosen solution through cross-model dimensional mapping, collinearity diagnostics, and phenotypic association profiling to evaluate whether the selected solution represented the optimal dimensionality.

#### 2.2.4. Stability and Reproducibility Assessment

Given the weakly supervised nature of Surreal-GAN, its output consists of multidimensional continuous variables that lack ground-truth labels for computing conventional accuracy or error metrics, making it impossible to directly assess model generalizability or stability through a single quantitative measure. To this end, we implemented a multi-stage validation strategy to ensure the reproducibility of the identified R-indices:

Consistency across repeated runs: To assess the impact of random initialization on the identification of aging patterns, we repeatedly trained the Surreal-GAN model under different random initialization conditions and verified the stability of the identified patterns against specific random effects by calculating the model consistency metric *R*_indices_corr_ (Section 2.2.3).

Cross-sample reproducibility: To assess whether the identified aging patterns are dependent on a particular training sample, we retrained the Surreal-GAN model on reproducibility set using identical hyperparameters. The new model was then applied to the reserved set, yielding new R-indices. The resulting R-indices were aligned with those from the original model via optimal assignment. High correlation would indicate that the aging dimensions are reproducible.

Cross-sex reproducibility: To evaluate the cross-sex reproducibility of the identified aging patterns, we split the original training set by sex into two mutually exclusive subsets (male and female) and independently retrained sex-specific Surreal-GAN models on each subset using the same hyperparameters as the original model. The male-specific model was then applied to the male subgroup of the reserved set, and the female-specific model to the corresponding female subgroup, yielding sex-specific R-indices. Pattern consistency was quantified by calculating the R-index correlation between the sex-specific models and the original model within the corresponding sex subgroups; a high correlation would indicate that the identified aging patterns are reproducible across sex groups.

### 2.3. Statistical Analysis

Post-development, the optimal model was applied to the combined application cohort, with subsequent downstream analyses structured at two complementary hierarchical levels. First, the neuroanatomical features of each aging pattern were topographically characterized within the reserved set, thereby establishing a clinically defined aging reference that is less confounded by disease-related variation. Subsequently, the analytical scope was broadened to the entire combined application cohort (incorporating both the reserved set and the non-CDAR TAR participants) to examine the associations of these aging patterns with multidimensional phenotypes across a clinically heterogeneous spectrum, and to systematically evaluate the extent to which different disease states are associated with deviations of individual brain aging patterns from this reference.

#### 2.3.1. Anatomical Characteristics of the Aging Patterns

The analyses in this part were conducted on the reserved set to characterize the neuroanatomical features of each aging pattern in clinically defined individuals. Before examining these neuroanatomical features, we first tested whether the identified aging patterns carried neuroanatomical information beyond chronological age, thereby evaluating the possibility that they merely reflected a redundant age axis or arose from a low-dimensionality preference. Specifically, we performed a nested model comparison in the reserved set, with age, sex, ICV, and education as baseline predictors, and assessed whether the inclusion of the aging patterns improved model fit using ΔR^2^, AIC reduction, and Bonferroni-corrected likelihood-ratio tests. Following this validation, the neuroanatomical features of each aging pattern were then further investigated using generalized linear models (GLM), adjusting for sex, age, ICV, educational attainment, and the other R-indices. Given that Surreal-GAN permits correlations among R-indices, entering the resulting R-indices simultaneously into a single model may introduce multicollinearity. To this end, the variance inflation factor (VIF) was employed as a diagnostic measure to evaluate the degree of collinearity among the R-indices. Using VIF < 5 as the threshold, values below this cutoff indicate that the model is free from severe multicollinearity and that the coefficient estimates possess acceptable stability. The regression equation was specified as follows:

(1) $$ROI=\beta_{0}+\beta_{t}\cdot R_{t}+\sum_{k\neq t}\beta_{k}\cdot R_{k}+\beta_{age}\cdot Age+\beta_{sex}\cdot Sex+\beta_{edu}\cdot Education+\beta_{ICV}\cdot ICV+\varepsilon$$

where $\beta_{t}$ is the regression coefficient for the target aging pattern, $R_{t}$ denotes the target aging pattern under analysis, and $\sum_{k\neq t}\beta_{k}\cdot R_{k}$ represents the other aging patterns, which serve as control variables. To facilitate comparison of effect sizes across different models and brain regions, the original coefficients were standardized:

(2) $$\beta_{std}=\beta_{t}\cdot \frac{SD(R_{t})}{SD(ROI)}$$

where $SD(R_{t})$ and SD(ROI) represent the sample standard deviations of the target aging pattern score and the brain regional measure, respectively. Because the derived R-indices capture distinct yet partially overlapping aspects of normative brain aging, their simultaneous inclusion in the GLM allows the regression coefficients to estimate the independent and complementary contributions of each pattern. Consequently, positive associations between specific patterns and regional volumes should be interpreted as relative preservation of regional brain structure, rather than absolute tissue growth.

For each aging pattern, we report the standardized regression coefficient (β) together with its 95% confidence interval. We characterized the anatomical features of each pattern through two complementary analyses: one based on the 139 ROI volumes derived from FSL’s FAST segmentation tool, and the other using cortical surface area and cortical thickness from the DKT atlas to supplement the volume-based findings with finer-grained morphometric detail.

#### 2.3.2. Analysis of Factors Associated with Brain Aging

All analyses in this section were conducted in the combined application cohort using GLM, with age, sex, and years of education included as covariates. Variables were analyzed using models appropriate to their type: for continuous variables, linear regression, with effect sizes reported as standardized β coefficients and *p* values; for count variables, the dispersion parameter was first calculated as the ratio of the Pearson chi-square statistic to the residual degrees of freedom, and a likelihood ratio test was used to compare Poisson and negative binomial models. If the dispersion parameter exceeded 1.5 and the likelihood ratio test was significant, negative binomial regression was adopted; otherwise, Poisson regression was used. Effect sizes were expressed as incidence rate ratios (IRR), accompanied by *p* values; for binary variables, binary logistic regression, with effect sizes reported as odds ratios (OR) and *p* values; and for ordinal variables, ordinal logistic regression, with effect sizes likewise reported as OR and *p* values. To control the false positive rate across multiple comparisons, the Bonferroni correction was applied, with the significance threshold set at corrected *p* < 0.01 (corresponding to an uncorrected *p* = 3.82 × 10^−5^). All statistical analyses were performed in R (version 4.3.3).

#### 2.3.3. Association with Disease Diagnoses

To investigate whether the identified aging patterns are involved in the pathogenesis of different diseases, we defined the following disease groups among non-CDAR TAR participants based on ICD-10 codes in the UKB: diabetes, hypertension, ischemic heart disease (IHD), chronic obstructive pulmonary disease (COPD), stroke, mental disorders, and neurodegenerative diseases. Individuals with multiple conditions were included in each applicable disease group; the reserved set was included as the clinically screened healthy control group (HC).

Given the limited sample size for certain specific diseases, we adopted a pragmatic grouping strategy, whereby disorders were grouped into broader categories to ensure stable statistical estimation. Specifically, neurodegenerative diseases encompassed Alzheimer’s disease (AD), Parkinson’s disease (PD), multiple sclerosis (MS), and various forms of cognitive impairment; mental disorders included depression, bipolar disorder, schizophrenia, anxiety disorders, and obsessive–compulsive disorder, among others. We acknowledge that this approach sacrifices disease specificity and may obscure heterogeneity among disease subtypes; therefore, the observed effect sizes should be interpreted as category-level associations rather than disease-specific estimates. In addition, UKB participants are predominantly community-dwelling individuals with well-controlled conditions; thus, observed effect sizes may be smaller than those in more severely affected clinical populations.

Multiple linear regression was employed, with age and sex included as covariates, to estimate the net effect of disease status on each aging pattern. The Bonferroni correction was applied to control for multiple comparisons, with the significance threshold set at *p* < 0.01. The regression equation was specified as follows:

(3) $$R=\beta_{0}+\beta_{age}\cdot Age+\beta_{sex}\cdot Sex+\sum_{d=1}^{7}\beta_{d}\cdot {Disease}_{d}+\varepsilon$$

where $\beta_{d}$ is the regression coefficient for disease, d representing the average difference in R-index scores between the diseased group and the HC after adjusting for age, sex, and all other diseases. The magnitude of the actual disease effect was assessed using Cohen’s *d*.

(4) $$d=\frac{\beta_{d}}{\sqrt{MSE}}$$

where MSE is the mean squared error of the model and $\sqrt{MSE}$ is the residual standard deviation.

## 3. Results

### 3.1. Model Selection

**Figure 2:**
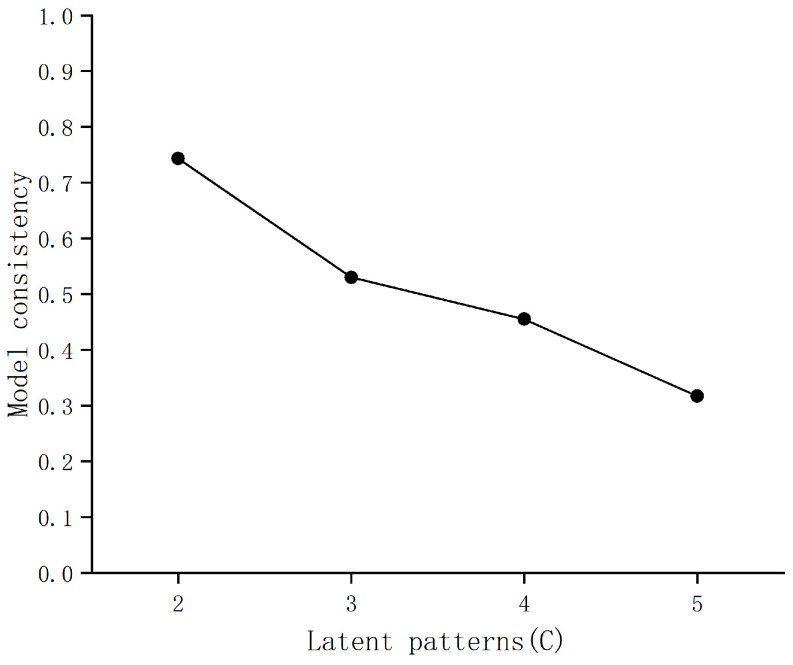
Sensitivity analysis of latent pattern (*C*).

To determine the number of patterns *C*, we fixed all other hyperparameters at their default values and systematically evaluated *C* in {2,3,4,5}. For each value of *C*, we repeatedly trained the model 20 times on the training dataset and calculated the *R*_indices_corr_ between models to robustly quantify solution reproducibility. The results showed that model consistency was highest at *C* = 2 and decreased monotonically as *C* increased (Figure 2). Accordingly, *C* = 2 was selected as the most reproducible solution under the current analytical framework. As described in Section 2.3.1, additional analyses were performed to evaluate whether the selected solution captured meaningful neuroanatomical information beyond chronological age. With *C* fixed at 2, the remaining regularization hyperparameters *λ* and *γ* were then tuned via grid search, with each hyperparameter combination trained independently 20 times; the highest *R*_indices_corr_ was obtained when *λ* = 0.05 and *γ* = 0.1 (Table 1). Accordingly, the final hyperparameter configuration was set to *C* = 2, *λ* = 0.05, and *γ* = 0.1. Under this configuration, the model was trained independently 40 times to assess the stability of the learned patterns and the robustness of the R-indices across repeated runs, yielding a final *R*_indices_corr_ of 0.736. For clarity, the 0.764 reported in Table 1 represents the best R_indices_corr_ for this hyperparameter combination during the grid-search phase (20 runs), whereas the 0.736 here corresponds to the best R_indices_corr_ from the final 40-run training; as they were derived from separate training phases, their difference reflects normal between-run variability in GAN retraining and is not directly comparable. The cross-sample reproducibility analyses reported in Section 3.4.1 further confirm the structural stability of the learned patterns. To rule out potential dimensional underestimation, we explicitly evaluated a higher-dimensional alternative model. Our exploratory findings demonstrated that expanding the model dimensions resulted in statistical over-partitioning of the existing variance and high phenotypic redundancy rather than uncovering meaningful biological heterogeneity, thereby confirming the two-dimensional solution as the optimal latent architecture (see Supplementary Table S4 for details). The trained model was subsequently applied to the combined application cohort to characterize the anatomical features of each aging pattern and to examine the associations with clinical, lifestyle, and multidimensional phenotypic factors.

### 3.2. Neuroanatomical Characterization of Aging Patterns in the Reserved Set

Using the optimized Surreal-GAN framework, two distinct GM aging patterns were identified from the reserved set. The nested model comparison showed that the inclusion of the identified aging patterns significantly improved model fit for the 139 regional brain volumes: the mean ΔR^2^ was 0.049 (range: 0.0004–0.514), 87.8% of regions passed the Bonferroni-corrected likelihood-ratio test (*p* < 0.01), and AIC decreased in 99.3% of regions. These results indicate that the two-dimensional solution carries substantive neuroanatomical information beyond chronological age, providing additional support for the biological interpretability of the identified patterns. Collinearity diagnostics showed that the VIF for each aging pattern was 1.69, which is well below the commonly used threshold (VIF < 5), indicating no serious collinearity between them and allowing both to be included in the multivariable regression model simultaneously. On this basis, to comprehensively map the neuroanatomical footprints of each axis, their associations with regional brain measures were examined, with all statistical significance thresholds strictly Bonferroni-corrected for multiple comparisons. FSL-FAST volumetric associations and the direction and count of significant cross-pipeline DKT associations are summarized in Table 2.

**Figure 3:**
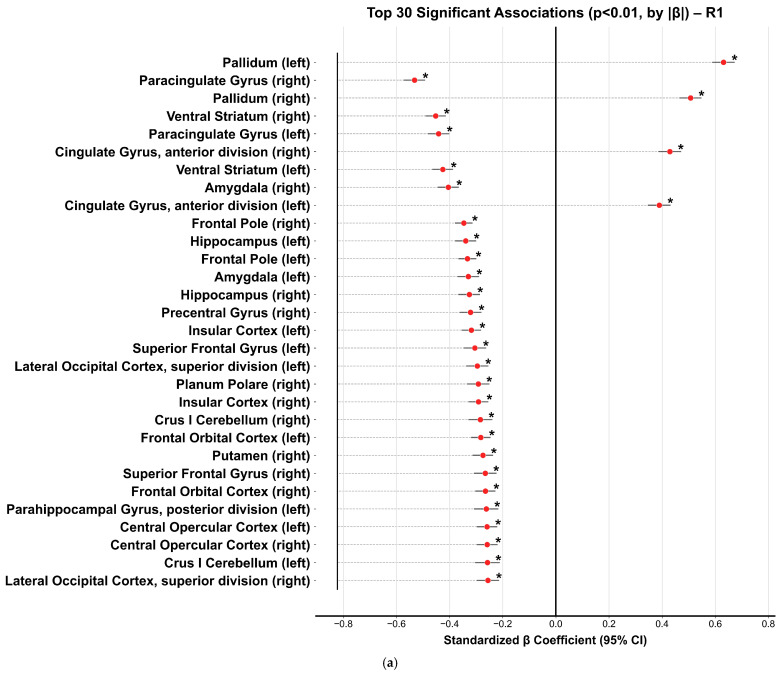

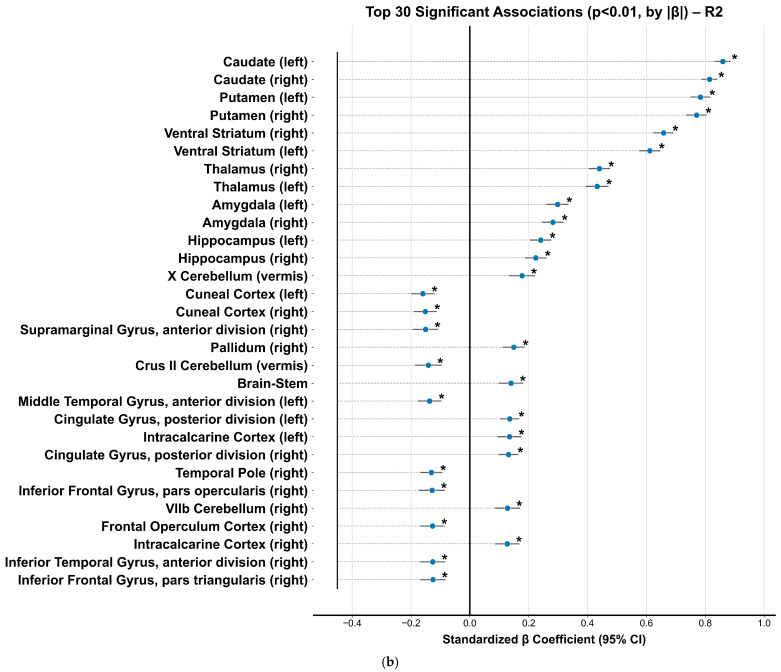
Association of GM aging patterns with regional brain volumes. This figure illustrates the statistical relationships between the decoupled aging patterns R_1_ and R_2_ and the regional brain volumes derived from FSL-FAST segmentation. For clarity, the 30 regions showing the strongest significant associations are displayed for each pattern: (**a**) R_1_ in red and (**b**) R_2_ in blue. Each point in the figure represents the standardized β coefficient between a given aging pattern and a specific brain region, with error bars indicating the corresponding 95% confidence interval. The formal definition and calculation workflow for the standardized coefficients are provided in Section 2.3.1. Asterisks highlight regions that strictly survive Bonferroni correction for multiple comparisons.

**Figure 4:**
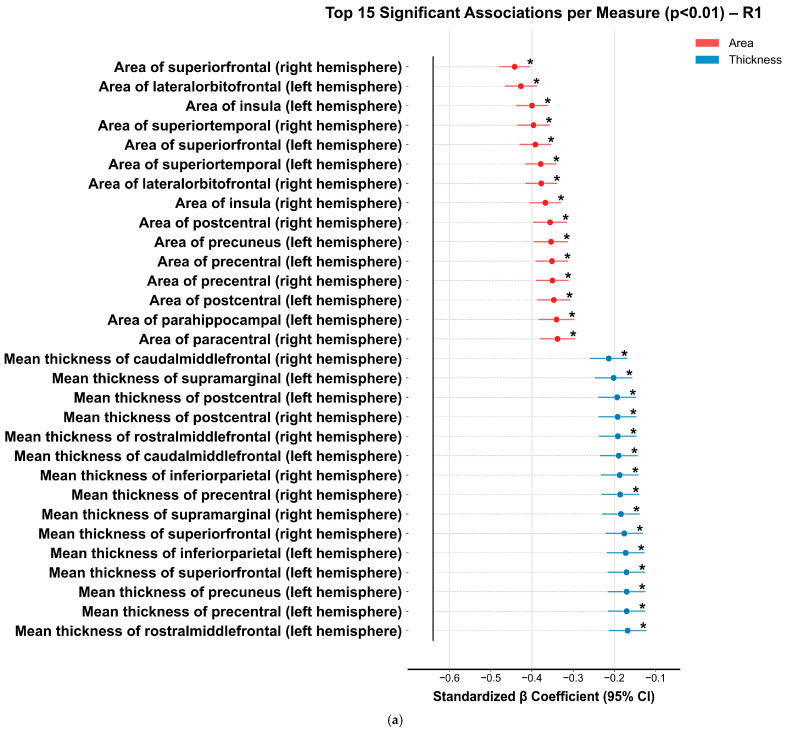

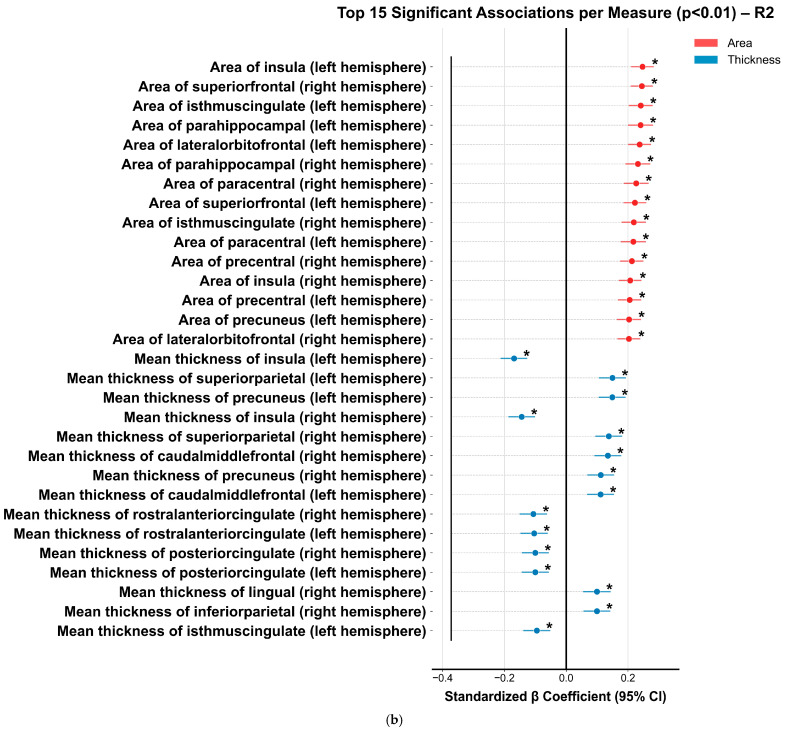
Association of aging patterns with surface area and cortical thickness. This figure illustrates the statistical relationships of the two decoupled aging patterns with morphometric metrics based on the DKT atlas, stratified into cortical surface area and cortical thickness. The top 15 regions with the most pronounced associations are shown separately for surface area (red) and cortical thickness (blue), with (**a**) displaying significant regions for R_1_ and (**b**) for R_2_. Each point in the figure represents the standardized β coefficient between a given aging pattern and a specific brain region, with error bars indicating the corresponding 95% confidence interval. Asterisks mark regions that remain significant after Bonferroni correction.

The two aging patterns showed divergent topographic profiles across the 139 FSL-FAST regional brain volumes. R_1_ was characterized by widespread negative associations spanning cortical, subcortical, and cerebellar territories, accompanied by selective positive associations in the anterior cingulate cortex and pallidum. In contrast, R_2_ exhibited a more spatially differentiated profile, characterized by positive associations with subcortical and limbic structures—including the caudate nucleus, putamen, ventral striatum, thalamus, hippocampus, and amygdala—with hemispheric asymmetry in the pallidum, and by regionally selective, mixed positive and negative associations in the cortex and cerebellum. This divergence was further characterized using cortical morphometric measures derived from the FreeSurfer DKT atlas as a cross-pipeline consistency check. R_1_ was dominated by a near-universal pattern of negative surface area associations, whereas R_2_ was characterized by widespread positive surface area associations alongside spatially selective and directionally heterogeneous changes in cortical thickness. Representative key regional results for R_1_ and R_2_ are presented in Figure 3 and Figure 4. Detailed statistical results are presented in Supplementary Table S2.

### 3.3. Symmetry Analysis of Hemispheric Aging Effects

To evaluate whether the learned aging-related representation captures system-level bilateral structural variation rather than hemispherically biased effects, we investigated the topographical symmetry of R-index–associated effects across the left and right cerebral hemispheres. Specifically, standardized β coefficients derived from regional brain volume measures were compared between left–right homologous regions using paired *t*-tests. The results revealed no significant global hemispheric differences in aging effects for either GM aging pattern (R_1_: *p* = 0.342; R_2_: *p* = 0.680).

To complement hemisphere-wise mean comparisons, which assess global differences in effect magnitude, we further evaluated the similarity of spatial distribution patterns between hemispheres. For each aging-related pattern, we constructed paired vectors of regional β for homologous regions in the left and right hemispheres, and quantified their similarity using Pearson correlation. The results showed a high degree of spatial correspondence between hemispheres for both patterns (R_1_: r = 0.963, 95% CI 0.941–0.978; R_2_: r = 0.981, 95% CI 0.968–0.988; both *p* < 0.001). The identified patterns were associated with broadly distributed bilateral associations rather than hemisphere-specific regional effects.

### 3.4. Latent Pattern Stability Analysis

#### 3.4.1. Cross-Sample Latent Pattern Correspondence

The model was retrained on the reproducibility set using the same hyperparameters as the original model, and the resulting new model was applied to the reserved set (ensuring the pattern estimation was performed on a low-confounding reference cohort), yielding a set of new aging patterns. Because Surreal-GAN is a weakly supervised deep generative model, its learned latent components are subject to standard identifiability indeterminacies, including permutation invariance and sign ambiguity, and therefore retraining may lead to reordering and polarity inversion of latent components rather than structurally distinct solutions. To address this, correspondence between original and retrained patterns was established as a label alignment problem under identifiability constraints, using Pearson correlation coefficients between the original R-indices and the new R-indices. Matching was restricted to one-to-one assignments consistent with maximal spatial similarity and was used solely to resolve permutation ambiguity rather than to optimize model agreement. Pearson correlation analyses were performed in IBM SPSS Statistics (version 27.0.1.0). The results showed a mean correlation coefficient of 0.913 (R_1_: 0.873, R_2_: 0.953).

#### 3.4.2. Subgroup-Based Pattern Stability Analysis (Sex-Stratified Split)

The train set was split into two mutually exclusive subsets (female and male). Models were independently trained on each subset using identical hyperparameters as the full model. Pearson correlation coefficients were evaluated following the same approach described above. The results showed strong correlations between the sex-specific models and the original aging patterns (mean correlation coefficient: female = 0.792, male = 0.818), indicating that the learned representations are robust to sex-based sampling variation within the training data.

### 3.5. Analysis of Associated Factors in the Combined Application Cohort

We first examined the associations of age, sex, and educational attainment with each aging pattern. The results showed that age was significantly associated with both R_1_ and R_2_, whereas sex and educational attainment were significantly associated with R_1_ (see details in Table 3). To account for the potential confounding effects of these variables, age, sex, and years of education were included as covariates in subsequent analyses where applicable.

In the cognitive domain, R_1_ and R_2_ showed different degrees of cognitive associations. Specifically, R_1_ was associated with widespread cognitive impairment, spanning multiple indices across reasoning, memory, processing speed, and executive function. By contrast, R_2_ exhibited a more restricted pattern of associations, with significant but weaker effects limited to executive function, processing speed, and select memory indices. In the lifestyle domain, R_1_ was significantly associated with adverse lifestyle profiles, including higher tobacco and alcohol consumption and slower usual walking pace, whereas R_2_ was significantly associated only with tea intake. Analysis of physical measures revealed that both patterns were significantly associated with cardiovascular health, respiratory function, physical fitness, and bone mineral density, although the strength of effects differed: R_1_ showed stronger effects than R_2_ on respiratory function and physical fitness, whereas R_2_ exhibited stronger effects than R_1_ on bone mineral density. Among all hematological markers, R_2_ was not significantly associated with any index, whereas R_1_ primarily showed abnormalities in markers related to glucose metabolism and liver function, as well as significant associations with inflammatory and erythroid cell markers. Beyond the above factors, R_1_ was significantly associated with social support and birth weight, while R_2_ was significantly associated with mental health. Detailed results are presented in Table 4 and Table 5. Given the large number of variables examined, these analyses are exploratory.

### 3.6. Disease Risk Association

**Figure 5:**
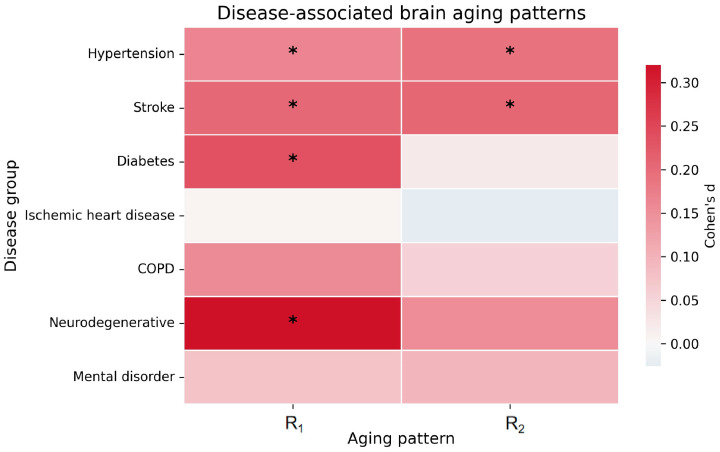
Associations between aging patterns and disease diagnoses. Warmer colors indicate larger Cohen’s d values; “*” denotes statistical significance after Bonferroni correction (*p* < 0.01); unmarked values indicate no significance correction.

Both aging patterns showed significant but modest associations with multiple diseases (Cohen’s d ranging from 0.165 to 0.32). Hypertension (R_1_: d = 0.165, R_2_: d = 0.188) and stroke (R_1_: d = 0.203, R_2_: d = 0.207) were significantly associated with both patterns. In addition, R_1_ showed associations with the overall burden of neurodegenerative conditions (d = 0.320) and diabetes (d = 0.235). All the above results remained significant after Bonferroni correction, as shown in Figure 5. Although the effect sizes were modest, these associations, given the stringency of the Bonferroni correction and the restricted range of the clinically defined reference cohort, may reflect subclinical vulnerability to disease associated with the aging patterns. To further examine the robustness of the disease associations, we conducted a sensitivity analysis, additionally adjusting for ICV, BMI, smoking status, and education (full results are provided in Supplementary Table S3). The results showed that the main disease associations remained robust after full covariate adjustment; only the association between R_2_ and stroke was no longer significant at the *p* < 0.01 threshold after adjustment, but remained significant at the *p* < 0.05 threshold, with the direction of effect unchanged, further supporting the reliability of the findings. Nevertheless, because the significance of some associations was attenuated after covariate adjustment, these disease association analyses should be interpreted as exploratory. Table 6 presents the demographic characteristics of each group.

## 4. Discussion

The heterogeneity of brain aging has been extensively investigated. However, previous studies using generative models such as GANs have primarily examined heterogeneous populations containing healthy individuals, disease states, and subclinical conditions, revealing disease-related atrophy patterns by contrasting patients with healthy controls. Consequently, the healthy control group has typically been treated as a relatively homogeneous reference group. By shifting the focus from disease heterogeneity to variation within this clinically screened cohort, we identified two dissociable but co-occurring neuroanatomical dimensions of brain aging. This core finding challenges the homogeneity assumption, demonstrating that a well-constrained reference space remains structurally heterogeneous rather than following a single neuroanatomical pattern. The dimensions identified here are interpreted as dominant modes of neuroanatomical variation within a clinically defined and low-confounding aging reference space, rather than universal axes of physiological brain aging. Table 7 summarizes the contrast in anatomical, cognitive, and systemic features associated with R_1_ and R_2_, to facilitate comparison between the two dimensions.

### 4.1. R_1_ Captures the Dominant Dimension of Age-Associated Structural Variation

Among the two aging dimensions, R_1_ emerged as the dominant, diffuse axis of age-related neuroanatomical variation, characterized by widespread cortical, subcortical, and cerebellar volume loss. A notable feature of the R_1_ topography was the preferential involvement of higher-order association cortices, accompanied by relative sparing of primary sensory regions, particularly the visual cortex. This spatial pattern aligns with the concept of association cortex vulnerability [35,36], suggesting that heteromodal cortical regions are disproportionately susceptible to aging-related structural decline and may reflect common organizational principles of cortical vulnerability [37]. It is also consistent with the retrogenesis framework [35] and the sensory-to-association gradient described in aging studies [38,39]. Cross-pipeline analyses revealed a coordinated pattern of negative associations involving both cortical surface area and cortical thickness, consistent with the Radial Unit Hypothesis [40,41], whereby surface area is largely established during prenatal neurogenesis, whereas cortical thickness is additionally shaped by lifelong processes.

Beyond the cerebral cortex, R_1_ featured synchronized volume reductions across the hippocampus, thalamus, striatum, and cerebellum, indicating a disruption of large-scale interconnected neural systems rather than isolated regional atrophy. This system-level pattern is further reinforced by extensive cerebellar involvement, consistent with its established role in cerebello-cortical executive circuits [42]. Within these widespread subcortical patterns, the anomalous preservation of the globus pallidus likely represents a technical artifact: age-related non-heme iron accumulation shortens T1 relaxation times, producing false hyperintensity that confounds automated segmentation [43]. Overall, R_1_ recapitulates the canonical neuroanatomical features consistently observed in normative brain aging.

To evaluate the functional relevance of the identified neuroanatomical patterns, we examined their relationships with cognitive performance. Behavioral analyses demonstrated that higher expression of R_1_ was associated with broad impairments across multiple cognitive domains, including memory, fluid reasoning, executive function, and processing speed. Given the established roles of the affected regions in cognitive control, memory processing, and large-scale information integration, these widespread structural alterations may help explain the broad cognitive deficits associated with R_1_. The present findings do not establish a direct causal pathway between structural alterations and cognitive outcomes. Instead, they indicate that the cross-hierarchical anatomical deterioration captured by R_1_ is associated with broadly reduced cognitive performance, linking widespread neuroanatomical vulnerability to behavioral heterogeneity in later life.

Beyond localized neuroanatomical variations, R_1_ scores exhibited robust associations with peripheral biomarkers, including elevated glycated hemoglobin (HbA1c), fasting blood glucose, gamma-glutamyl transferase (GGT), neutrophil counts, and total white blood cell counts. This coupling between widespread brain atrophy and peripheral physiological dysregulation suggests that R_1_ captures the cerebral expression of broader inflammaging and metabolic aging processes. Such physiological dysregulation may converge on the neurovascular unit [44], contributing to structural decline. These biomarkers are collectively consistent with chronic metabolic, oxidative, and inflammatory stress, potentially impairing neurovascular coupling and microvascular integrity. Given their high metabolic demands and dense vascular support, the transmodal association cortices and subcortical systems affected by R_1_ may be particularly vulnerable to age-related vascular and metabolic decline.

The clinical relevance of R_1_ was further evaluated by examining its associations with age-related systemic diseases. Individuals with higher R_1_ expression exhibited increased prevalence of cardiometabolic and neurodegenerative conditions. Importantly, these associations do not imply that R_1_ represents a prodromal disease state or a direct precursor of any specific clinical diagnosis. Rather, R_1_ may reflect a structural aging state associated with increased vulnerability to adverse health outcomes, consistent with neuroanatomical features frequently reported across multiple age-related disorders [45,46]. Accordingly, it provides a macroscopic neuroanatomical perspective for understanding heterogeneity in late-life health outcomes.

### 4.2. R_2_ as a Structural Preservation Pattern Associated with Brain Aging

Beyond the classic aging pattern captured by R_1_, we identified a second, qualitatively distinct aging pattern within this clinically defined operational reference cohort—one that coexisted with R_1_ but was dissociable from it. The morphometric profile of R_2_ contrasted sharply with the diffuse negative association in R_1_. R_2_ was characterized by widespread positive associations with cortical surface area alongside regionally heterogeneous associations with cortical thickness. As noted above, these two morphometric properties have distinct developmental origins. Accordingly, the broad preservation of cortical surface area may reflect maintenance of early-established cortical architecture, whereas the heterogeneous thickness profile may reflect region-specific lifelong tissue remodeling. Together, these findings support the conceptualization of R_2_ as an aging dimension characterized by relative structural preservation despite age-associated variation.

Intriguingly, R_2_ exhibited a regionally differentiated pattern of cortical thickness associations, characterized by relative preservation of posterior parietal regions, including the bilateral precuneus and superior parietal lobule, together with selective thickness preservation of frontal regions such as the caudal middle frontal gyrus, whereas thickness reductions were observed in the bilateral insula, rostral anterior cingulate cortex, and posterior cingulate cortex. This regionally selective pattern is broadly consistent with the Scaffolding Theory of Aging and Cognition-revised (STAC-r) [47], suggesting differential preservation of cortical systems involved in executive control, spatial cognition, and large-scale network integration, rather than uniform maintenance across the cortex.

A central question is whether this apparent preservation should be interpreted within a compensatory framework or a maintenance framework. Individuals with higher R_2_ expression exhibited relative preservation of cortical surface area across much of the cortex, together with preservation of several key subcortical structures, including the caudate nucleus, putamen, thalamus, hippocampus, and amygdala. Based on this structural profile, the present findings are more consistent with the Brain Maintenance hypothesis, which argues that successful cognitive aging primarily reflects the preservation of structural and functional brain integrity rather than enhanced compensatory recruitment [48,49]. Although the absence of functional measures precludes definitive conclusions about compensatory processes, the structural profile of R_2_ is consistent with relatively preserved neural substrates supporting cognitive performance despite advancing age. Accordingly, R_2_ may represent an aging dimension characterized by relative structural preservation, although confirmation of a maintenance rather than compensatory mechanism will require functional evidence.

Phenotypic analyses further revealed distinct systemic association patterns for R_1_ and R_2_, whereas R_1_ showed robust relationships with multiple markers of metabolic dysfunction and chronic low-grade inflammation, R_2_ demonstrated little or no association with most of these indicators, suggesting that the two dimensions capture partially distinct biological processes rather than different degrees of the same aging trajectory. Although the biological basis of R_2_ remains uncertain, its widespread preservation of cortical surface area may reflect developmental reserve established early in life, whereas preserved subcortical hubs may indicate maintenance of large-scale neural network organization.

### 4.3. From Mixed Aging–Disease Heterogeneity to Intrinsic Aging Dimensions

Taken together, R_1_ and R_2_ provide a direct answer to the central question of this study: even when overt disease-related variance is minimized through stringent clinical screening, brain aging does not follow a single pattern, but exhibits structured heterogeneity in the form of two dissociable dimensions. Previous Surreal-GAN analyses identified multiple latent dimensions, several of which were strongly associated with AD biomarkers and clinical progression, indicating substantial enrichment for disease-related pathological variance [22]. In contrast, the present study focused on a clinically screened CDAR cohort, in which disease-related neuroanatomical variation was substantially reduced. Rather than emphasizing the number of latent dimensions, our primary question was whether structured heterogeneity remains after minimizing disease-related confounding.

In terms of spatial distribution, the identified R_1_ dimension, characterized by diffuse cortical, subcortical, and cerebellar atrophy, demonstrates substantial spatial concordance with the cortical thinning patterns of the original R_4_ dimension (diffuse cortical atrophy), while extending these shared features into broader subcortical and cerebellar systems. This reduction in disease-related heterogeneity may contribute to reshaping the learned latent structure, allowing diffuse age-related neuroanatomical variation to emerge as the dominant latent dimension and facilitating the identification of preservation-related patterns. Previous brain aging models have predominantly captured structural decline and disease-related neuroanatomical variation, whereas a distinct latent dimension reflecting relative structural preservation has been less well characterized. In the present context, ‘structural preservation’ refers specifically to relatively above-average regional maintenance of grey matter volume for a given age, reflecting heterogeneity in the rate and extent of age-related tissue loss rather than the absence of atrophy. Because disease-related structural changes generally exceed the magnitude of normal age-related changes, they typically dominate the latent space and obscure subtle, preservation-related trajectories [50,51]. By minimizing this disease-related heterogeneity, the present study reshapes the learned latent structure, allowing diffuse normative variation to emerge as the dominant axis while successfully unmasking the preservation-related pattern.

### 4.4. Limitations and Future Directions

This study has several limitations. First, UKB participants are predominantly White British and community-dwelling volunteers, which limits the generalizability of the identified neuroanatomical patterns and their associated factors to broader populations. Second, this study used only T1w MRI. Although such data can reliably characterize GM atrophy patterns, brain aging also involves WM microstructural changes and functional network reorganization. Future integration of multimodal data, such as diffusion MRI and functional MRI, would help to more comprehensively reveal the multidimensional features associated with aging in individuals without clinically diagnosed conditions. Furthermore, this study employed a cross-sectional design and therefore cannot verify whether the identified age-associated patterns correspond to longitudinal changes over time. For the same reason, although the nested model comparison, spatial specificity analyses, and external association profiles provide converging support for the biological interpretability of R_1_ and R_2_, definitive validation of their longitudinal relevance and biological interpretation awaits future longitudinal cohort data. Future longitudinal studies could clarify whether these two dimensions reflect dissociable longitudinal aging patterns.

At the methodological level, this study provides an operational reference framework for brain aging heterogeneity research within the CDAR cohort. The CDAR framework cannot fully exclude preclinical pathological burden. Subclinical vascular injury, preclinical neurodegenerative processes, medication effects, and silent proteinopathies may still contribute to the observed variance. Integrating the CDAR framework with molecular biomarkers in future work represents an important extension of this research direction. Finally, when exploring factors associated with the aging patterns, this study only reported statistical associations using generalized linear models without conducting rigorous causal inference. Due to the lack of causal validation methods, it remains challenging to definitively clarify the directional nature of these links. Subsequent research could introduce methods such as Mendelian randomization to further clarify the causal relationships between these factors and brain aging patterns.

## 5. Conclusions

Our study provides new insights into brain aging. By rigorously excluding overt clinical pathology, we found that brain aging in individuals free from major clinical conditions is not adequately characterized by a single neuroanatomical pattern, but instead manifests as two co-occurring dimensions: one characterized by diffuse cortical atrophy accompanied by broadly poorer cognitive performance and poorer functional profiles, and the other marked by relatively preserved subcortical structures with limited cognitive decline. These findings suggest that clinically screened populations exhibit structured neuroanatomical heterogeneity rather than a uniform pattern of age-associated brain variation. Moreover, this heterogeneity is associated with multiple non-pathological factors, including lifestyle habits and psychosocial factors. Overall, the findings support the view that the clinically screened cohort contains structured neuroanatomical heterogeneity and demonstrate how cohort design may influence the latent organization recovered by generative models. Future integration with longitudinal and multimodal data will be necessary to determine whether these dimensions correspond to longitudinal changes or represent stable patterns of age-associated variation across the lifespan.

## Figures and Tables

**Table 1:** Model consistency under different hyperparameter combinations.

	*λ* = 0.05	*λ* = 0.2	*λ* = 0.4	*λ* = 0.8
*γ* = 0.1	**0.764**	0.668	0.704	0.615
*γ* = 2	0.694	0.629	0.660	0.545
*γ* = 6	0.706	0.649	0.602	0.551

Note: Results were obtained under *C* = 2. For each hyperparameter combination, the model was trained independently 20 times, and the corresponding *R*_indices_corr_ is reported as the consistency metric. The optimal result is highlighted in bold.

**Table 2:** Summary of aging patterns and morphometric associations.

Pipeline	Measure	R-Index	Significant (n/N)	Direction (neg/Pos)
FSL-FAST	Volume	R_1_	97/139	89 neg/8 pos
		R_2_	65/139	28 neg/37 pos
FreeSurfer DKT	Surface area	R_1_	61/62	61 neg/0 pos
		R_2_	55/62	0 neg/55 pos
	Thickness	R_1_	40/62	40 neg/0 pos
		R_2_	16/62	7 neg/9 pos

Note: FSL-FAST volumetric measures comprised 139 regions. DKT surface area and cortical thickness each comprised 62 measures. Significance was determined after Bonferroni correction (*p* < 0.01). “Pos” indicates a positive association, and “neg” indicates a negative association.

**Table 3:** Associations between covariates and aging patterns.

	R_1_	R_2_
Age	<0.001 *	<0.001 *
Sex	<0.001 *	0.07
Education	<0.001 *	0.51

“*” indicates statistically significant results.

**Table 4:** Associations between phenotypes and aging patterns.

Dimension	Component	Variable	Type	Subjects	R_1_	R_2_
Lifestyle	Diet	Cereal intake	continuous ^1^	12,922	−0.058	— ^2^
		Tea intake	continuous	12,926	—	−0.047
	Smoking	Pack years of smoking	continuous	3709	0.077	—
	Alcohol intake	Mean weekly alcohol intake	continuous	9138	0.074	—
	Physical activity	Usual walking pace	ordinal ^3^	12,912	0.64	—
Physical measurements	Cardiovascular risk factors	Diastolic blood pressure	continuous	10,083	0.077	0.103
		Systolic blood pressure	continuous	10,080	0.067	0.072
		Pulse rate	continuous	10,083	0.077	0.091
		Hip circumference	continuous	12,646	—	−0.044
	Respiratory function	Forced vital capacity (FVC)	continuous	7509	−0.092	−0.046
		Forced expiratory volume in 1 s (FEV1)	continuous	7509	−0.084	−0.033
	Bone mineral density	Heel bone mineral density (BMD)	continuous	7061	−0.062	−0.082
		BMD T-score	continuous	7064	−0.06	−0.081
	Physical fitness	Hand grip strength (left)	continuous	12,576	−0.07	−0.038
		Hand grip strength (right)	continuous	12,580	−0.069	−0.035
Blood biochemistry	Glucose metabolism	Glycated haemoglobin (HbA1c)	continuous	12,149	0.087	—
		Glucose	continuous	11,142	0.06	—
	Liver function	Gamma glutamyltransferase	continuous	12,177	0.046	—
		Total protein	continuous	11,146	0.043	—
Blood count	Reticulocytes	High light scatter reticulocyte count	continuous	12,280	0.05	—
		Immature reticulocyte count	continuous	12,280	0.045	—
		High light scatter reticulocyte percentage	continuous	12,280	0.051	—
	White blood cell series	Neutrophil count	continuous	12,433	0.063	—
		Leukocyte count	continuous	12,453	0.048	—
Psychosocial factors	Social support	Able to confide	ordinal	12,431	0.757	—
	Mental health	Nervous feelings	binary ^4^	12,632	—	1.42
Early-life factors	Early life	Birth weight	continuous	6598	−0.106	—

Note: All numerical values shown in the table reached statistical significance at *p* < 0.01 after Bonferroni correction. Effect sizes are reported according to variable type. ^1^ Continuous variables; effect sizes are reported as standardized β coefficients, with values greater than 0 indicating positive associations and values less than 0 indicating negative associations. ^2^ “—” indicates non-significance after correction (*p* ≥ 0.01); non-significant variables are not listed. ^3^ Ordinal categorical variables; effect sizes are reported as OR, with values greater than 1 indicating positive associations and values less than 1 indicating negative associations. ^4^ Binary categorical variables; effect sizes are reported as OR, with values greater than 1 indicating positive associations and values less than 1 indicating negative associations.

**Table 5:** Associations between cognitive function and aging patterns.

Cognitive Domain	Cognitive Test	Variable	Type	Subjects	R_1_	R_2_
Non-verbal Reasoning	Matrix Pattern Completion	Number of puzzles correctly solved	count ^1^	8712	0.92	—
Verbal and numerical reasoning	Fluid Intelligence Test	Fluid intelligence score	continuous	11,611	−0.09	—
		Number of fluid intelligence questions attempted within time limit	count	11,611	0.93	—
Visual declarative Memory	Pairs Matching Test (Round 2)	Number of incorrect matches in round	count	11,655	1.13	1.08
		Time to complete round	continuous	11,655	0.06	—
Prospective memory	Prospective Memory Test	Correct recall on first attempt	binary	11,960	0.65	—
Executive function	Tower Rearranging Test	Number of puzzles correct	count	8587	0.9	—
		Number of puzzles attempted	count	8869	0.94	—
	Trail Making Test (Part B)	Total errors traversing alphanumeric path	count	8340	1.43	1.15
		Duration to complete alphanumeric path	continuous	8340	0.09	0.05
	Trail Making Test (Part A)	Duration to complete numeric path	continuous	8715	0.08	—
Processing speed	Symbol Digit Substitution Test	Number of symbol digit matches made correctly	count	8720	0.91	0.94
		Number of symbol digit matches attempted	count	8720	0.92	0.95
	Reaction Time Test	Mean time to correctly identify matches	continuous	11,845	0.05	—

^1^ Count variables; effect sizes are reported as IRR, with values greater than 1 indicating positive associations and values less than 1 indicating negative associations. For the annotation of other effect sizes, refer to Table 4. All numerical values shown in the table reached statistical significance at *p* < 0.01 after Bonferroni correction.

**Table 6:** Statistical descriptions of participants.

Group	Subjects	Age (Years)
HC	3398	70.03 ± 3.75
diabetes	1192	71.23 ± 3.85
hypertension	5923	71.82 ± 3.99
IHD	2183	71.84 ± 4.13
COPD	389	70.43 ± 3.76
stroke	328	70.96 ± 3.86
neurodegenerative diseases	289	71.30 ± 3.93
mental disorders	1185	71.18 ± 3.94

**Table 7:** Summary contrast between R_1_ and R_2_ aging patterns.

Feature Domain	R_1_	R_2_
Overall pattern	Diffuse atrophy	Relative structural preservation
Cortical volume	Widespread negative correlations (frontal, temporal, insular, and partial parieto-occipital)	Positive correlations in posteromedial cortex, insula, and occipital lobe; negative correlations in lateral convexity
Subcortical volume	Widespread negative correlations (hippocampus, thalamus, caudate, putamen, ventral striatum); positive correlation in pallidum	Positive correlations in caudate, putamen, ventral striatum, thalamus, hippocampus, and amygdala
Cerebellum	Widespread negative correlations (anterior and posterior lobes, and parts of vermis)	Mixed positive and negative, predominantly positive
Cortical surface area	Negative in all significant regions	Positive in all significant regions
Cortical thickness	Regionally distributed, with negative associations in all significant regions.	Regionally mixed (positive and negative)
Cognitive associations	Poorer performance across multiple domains (reasoning, memory, processing speed, executive function)	Weaker effects, limited to executive function, processing speed, and selected memory measures
Peripheral phenotypes	Associated with metabolism (HbA1c, glucose), liver function (GGT), and inflammation (neutrophils)	No significant metabolic–inflammatory associations
Lifestyle	Associated with smoking, alcohol consumption, and physical activity	Associated only with tea intake
Disease associations	Diabetes, neurodegenerative diseases, hypertension, stroke	Hypertension, stroke

## Data Availability

The imaging data analyzed in this study were obtained from the UK Biobank and are available through the UK Biobank Access Management System (http://www.ukbiobank.ac.uk/register-apply/; accessed on 20 September 2025). Access to these data is governed by the UK Biobank Research Access Administration Team and is open to both academic and commercial applicants under the same review procedures. Applications are assessed based on their relevance to health-related research objectives. Additional information regarding available datasets can be found on the UK Biobank website (http://www.ukbiobank.ac.uk; accessed on 20 September 2025). Owing to ongoing updates to the resource, the number of participants with available imaging data may differ slightly from the sample reported in this study. The Surreal-GAN framework is publicly available from Yang et al. (https://github.com/zhijian-yang/SurrealGAN, [22]; accessed on 20 September 2025). The hyperparameter configuration used in this study is fully reported in Section 2.2.3 Model Training and Hyperparameter Optimization and Section 3.1 Model Selection. Analysis code is available from the corresponding author upon reasonable request.

## Supplementary Materials

The following supporting information can be downloaded at: https://www.mdpi.com/article/10.3390/bioengineering13070844/s1, Table S1: ICD-10 exclusion codes used to define the clinically screened healthy CDAR cohort. Table S2: Detailed statistical results for associations between neuroimaging features and the two aging patterns, comprising: Table S2A. Detailed statistical results for associations between FSL-FAST regional brain volumes and the two aging patterns, and Table S2B. Detailed statistical results for associations between cross-pipeline DKT cortical surface area and thickness and the two aging patterns. Table S3: Sensitivity analysis for disease associations with full covariate adjustment. Table S4: Validation of the C = 3 solution, comprising: Table S4A. Associations of each pattern with peripheral phenotypes at C = 3, Table S4B. Associations of each pattern with cognition at C = 3, Table S4C. Cross-model dimensional mapping, Table S4D. Internal collinearity of the C = 3 split subcomponents, and Table S4E. Detailed statistical results for associations between each pattern and regional brain volumes at C = 3.
